# Charge Storage and Solar Rechargeable Battery Devices Based on Electrodes Electrochemically Modified with Conducting Polymer Nanowires

**DOI:** 10.3390/polym13244375

**Published:** 2021-12-14

**Authors:** Andrés Mauricio Ramírez, Manuel Alejandro Gacitúa, Fernando Raúl Díaz, María Angélica del Valle

**Affiliations:** 1 Laboratorio de Electroquímica y Materiales Aplicados, Centro de Nanotecnología Aplicada, Facultad de Ciencias, Universidad Mayor, Camino La Pirámide 5750, Santiago 8580745, Chile; andres.ramirez@umayor.cl; 2 Facultad de Química y Biología, Universidad de Santiago de Chile, Av. L.B. O’Higgins 3363, Santiago 7254758, Chile; manuel.gacitua@usach.cl; 3 Laboratorio de Electroquímica de Polímeros, Pontificia Universidad Católica de Chile, Av. V. Mackenna 4860, Macul, Santiago 7820436, Chile; fdiaz@uc.cl

**Keywords:** electrochemical polymerization, polymer nanowires, battery, solar rechargeable battery

## Abstract

In this work, the use of nanostructured conducting polymer deposits on energy-storing devices is described. The cathode and the anode are electrochemically modified with nanowires of polypyrrole and poly(3,4-ethylenedioxythiophene), respectively, prepared after the use of a mesoporous silica template. The effect of aqueous or ionic liquid medium is assayed during battery characterization studies. The nanostructured device greatly surpasses the performance of the bulk configuration in terms of specific capacity, energy, and power. Moreover, compared with devices found in the literature with similar designs, the nanostructured device prepared here shows better battery characteristics, including cyclability. Finally, considering the semi-conducting properties of the components, the device was adapted to the design of a solar-rechargeable device by the inclusion of a titanium oxide layer and cis-bis(isothiocyanate)-bis(2,2′-bipyridyl-4,4′-dicarboxylate) ruthenium (II) dye. The device proved that the nanostructured design is also appropriate for the implementation of solar-rechargeable battery, although its performance still requires further optimization.

## 1. Introduction

Recently, the use of conducting polymers has drawn attention in the development of energy storage devices by using the inherent doping/undoping process [1,2,3,4]. Poly-3,4-ethylenedioxide thiophene (PEDOT) and polypyrrole (PPy) have been used in the design of rechargeable batteries in past. For instance, Kim et al. [5] studied vanadate–PEDOT composite with Zn^2+^ insertion ion in rechargeable batteries, proving that 94% of total specific capacity is maintained after 1000 charge/discharge cycles. On this sense, PEDOT interaction with oxygen from vanadate prevents structural breakdown of the active layer, improving Zn^2+^ insertion. Additionally, Huang et al. [6] reported the use of PEDOT in a NiO composite for rechargeable batteries with Li+ as insertion ion; the authors proved that the use of the conducting polymer notably improves stability of the device.

The synthesis of nanostructured conducting polymer layers has also shown great improvement in several electrochemical devices such as fuel cells and sensors [7,8,9,10,11,12,13] due to the increased active surface offered by nanometric structures over electrodes. For instance, Schoetz et al. [14] made use of nanostructured PEDOT as cathode material in rechargeable ionic liquid-based batteries, showing good stability even at high charge/discharge rates. On the hand, Jung et al. [15] published the use of a composite between tin and nanostructured PPy as anode in Li-ion batteries improving ion diffusion. The electrochemical synthesis of PEDOT and PPy nanowires can be accomplished through the use of template, modifying the methodology described by Walcarius [16,17,18,19]. In a previous step, it is necessary to grow a thin film of the same polymer to guarantee the adhesion of these nanostructures to the substrate [20,21]. This method has made possible to obtain improved charge values of the doping/undoping process and better reversibility, using only electrochemical techniques [7,21].

Even though the use of conducting polymer has proven to be advantageous in battery development reports, the careful selection of both electrolyte and solvent have a key role in determining the overall performance of the device [20,22,23,24]. In this sense, ionic liquids exhibit advantages over dissolved electrolyte medium such as stability at high temperatures, not flammable, low vapor pressure, high ionic conductivity [25], and are an excellent alternative for the development of batteries [1,14].

In addition, an important contribution has been made by Liu et al. [26], who reported a solar rechargeable battery constructed using a TiO_2_|PEDOT photo-anode PPy counter electrode. The author described a device with rapid photo-recharge under illumination and a stable electrochemical discharge in the dark [26].

In present report, nanostructured PEDOT and PPy deposits are used for the design and characterization of energy storing devices and compared to analogous devices with bulk polymeric deposits. In addition, the outcomes present comparisons between the use of aqueous medium and ionic liquid during device manufacturing. Finally, the possibility of using the nanostructured electrodes on the assembly of a solar energy rechargeable battery is presented.

## 2. Materials and Methods

For all electrochemical measurements, a CH Instruments potentiostat/galvanostat (CH Instruments, Inc., Austin, TX, USA) is used, controlled by the CH750D software (version 11.13, CH Instruments, Inc., Austin, TX, USA), and the tests of the different batteries are carried out on an OrigaLys model OGF500 potentiostat/galvanostat (OrigaLys ElectroChem SAS, Lyon, Auvergne-Rhône-Alpes, France), in a charge/discharge module controlled by OrigaMaster software (version 2.2.0.1, OrigaLys ElectroChem SAS, Lyon, Auvergne-Rhône-Alpes, France).

The modified electrodes were prepared using the conditions previously reported in [7,21,27]. Briefly, the electrochemical polymerization was carried out in the three-compartment anchor cells, using a Ag|AgCl wire immersed in tetramethylammonium chloride solution that matched the potential of a saturated calomelane electrode [28] as reference and a Pt wire with high geometrical area as counter electrode. Working electrode corresponds to conductive glass with indium-doped tin oxide (ITO), previously washed for 5 min with ethanol and then 5 min in acetone to degrease. For conducting polymer nanowires deposition, the ITO electrode was previously modified with a mesoporous silica template by electrochemical method previously reported [29]. Nanostructured anode [27] and cathode [21] were prepared under tested optimized conditions. The deposited conducting polymer mass were calculated using the methodology proposed by del Valle et al. [20,22].

**Scheme 1:**
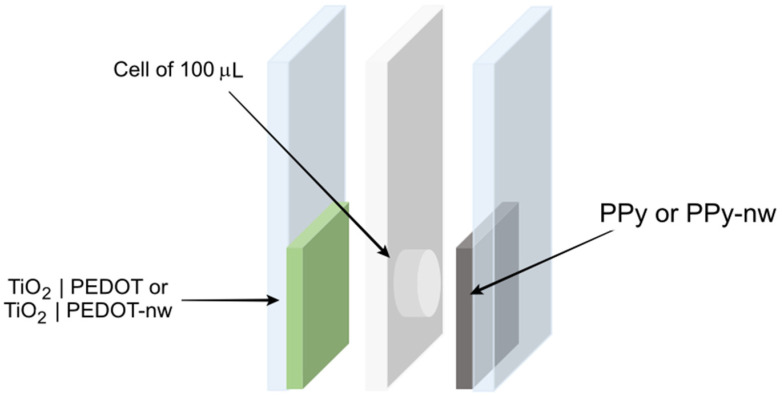
Two electrode cell construction design for measure of recharge and discharge.

The response of polymer-modified electrodes, based on their implementation in rechargeable and discharge batteries, was measured in 10^−1^ mol L^−1^ LiCl in milli-Q water and ionic liquid 1-butyl-3-methylimidazolium hexafluorophosphate (BMIMPF_6_) using different charge and discharge currents, ranging from 2.3 to 22.3 mA, Scheme 1. Finally, the preparation of a photo-rechargeable device was performed including a layer of N3 dye (cis-bis(isothiocyanate)-bis(2,2′-bipyridyl-4,4′-dicarboxylate) ruthenium(II)) [27] before PEDOT deposition at the anode. Solar rechargeable battery characterization was made in a Teflon cell, illuminated with 100 mW cm^−2^ white light.

## 3. Results and Discussion

### 3.1. Battery Characterization

**Figure 1:**
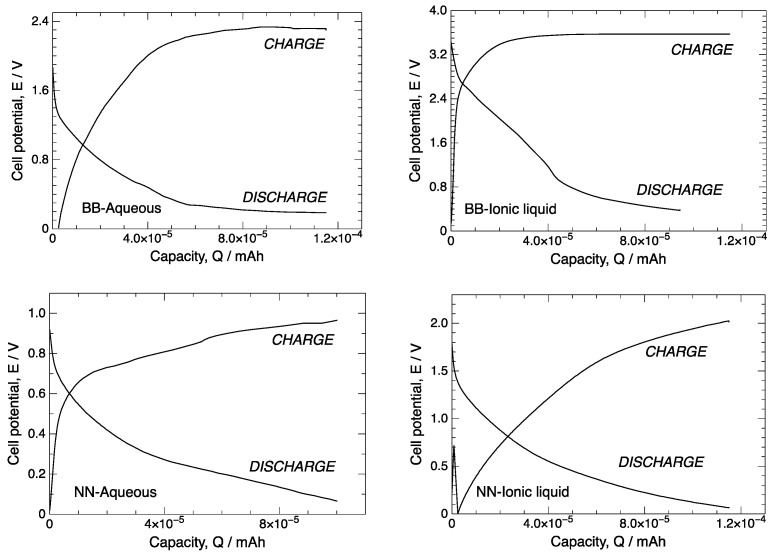
Characteristic charge and discharge cycle of PEDOT|PPy battery, charged with 2.3 μA and discharged with −2.3 μA.

The electrosynthesis of conducting polymers nanowires over the electrodes was accomplished presenting 30 nm diameter wires grown normal to the electrode surface (Figure S1, in Supplementary Information) as reported in past studies [7,21]. Conducting polymer modified electrodes are evaluated as a charge accumulator device, based on their respective *p*-doping/undoping (charge/discharge) processes. Bulk- (B, regular deposits without template) and nano- (N, nanowires prepared with silica template) polymeric deposits were prepared for PEDOT and PPy and combined for battery characterization. Bulk–bulk (BB) and nano–nano (NN) configurations were tested to check the effect of selected currents and media on single-step charge/discharge profile stability (Figure S2). The stability of the charge/discharge process is evaluated by transient smoothness and similarity of potential values reached at the end of each step. In general terms, all configurations and media show low stability at 16.7 μA or higher, with rough potential profiles and considerable potential asymmetry after charge and discharge steps. The low-stability transient may be caused by polymer over-oxidation at high currents, evidenced by PEDOT layers color shift from blue to brown. Nevertheless, a higher stability is reached by NN cells if compared to BB at least at 5 and 2.3 μA charge current. In fact, whether for aqueous or ionic liquid media at 5 μA current a multi-charging process takes places at BB [1], very different to that observed with NN. This can be attributed to the higher ramification degree of polymeric chains achieved during bulk electrochemical polymerization, while the use of template would generate a more uniform chain length by confining the growth nanopores space. Finally, at 5 μA the potential value reached by the BB almost doubles that obtained for the NN in each media. If both media are compared, in the presence of ionic liquid, the different systems have higher charge potentials; in BB and NN setups this is attributed to anion diffusion towards the anode–solution interface [30], caused by the viscosity of this non-aqueous medium [1]. With respect to device duration, a low and stable potential is desired; thus, 5 μA charge seems appropriate to test cyclability of the device. To evaluate battery characteristics, charge/discharge curves at ±2.3 mA for the BB and NN setups in different media are presented in Figure 1.

Typical battery parameters, such as specific total charge capacity *Q_tot_* (Ah g^−1^), discharge energy density (or specific energy) *E_spec_* (Wh kg^−1^), and discharge power density (or specific power) *P_spec_* (W kg^−1^) per mass of deposit m (g) were calculated through Equations (1)–(3) [14,31], as follows:

(1) $$Q_{tot}=\frac{Q_{c}\times Q_{a}}{Q_{c}+Q_{a}}$$

(2) $$E_{spec}=\frac{EIt}{m_{PEDOT}}$$

(3) $$P_{spec}=\frac{EI}{m_{PEDOT}}$$

where the maximum charge, *Q_c_*, and the maximum discharge capacities, *Q_a_*, (mAh g^−1^) are evaluated with the maximum capacity reached at Figure 1 divided by total mass of PEDOT and PPy deposited, respectively. The average discharge potential E (V) is the potential reached by the end of the discharge process, current I is 2.3 μA (2.3 × 10^−6^ A) and discharge time is 180 s (0.05 h). Battery characteristics are presented on Table 1.

**Figure 2:**
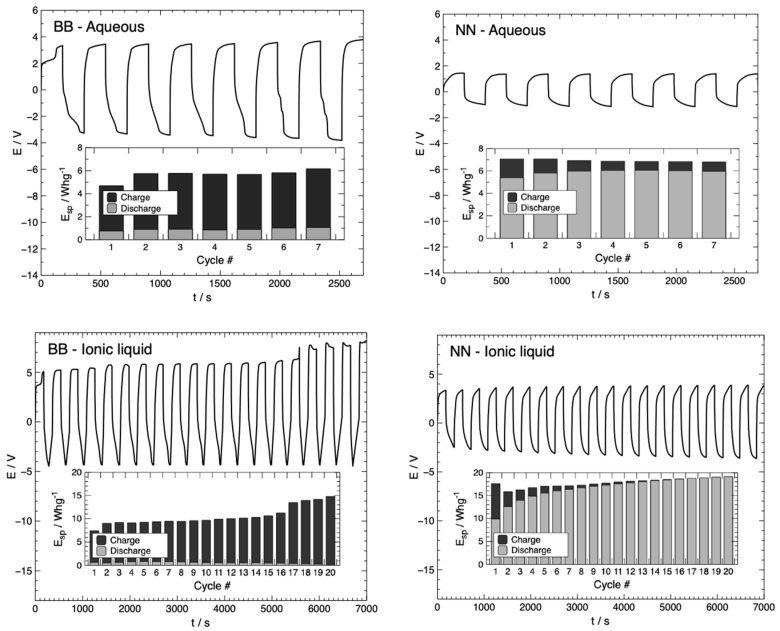
Multiple-steps E/t charge/discharge transients registered at ±5 μA for bulk (BB) and nanostructured (NN) electrode configuration in different media. Insert: Energy density calculated for each charge/discharge cycle.

The total specific capacity is ca. 16 times higher when using the NN configuration in both aqueous and ionic liquid media, which could be explained by the increased nanostructured electrode active surface area offered by nanowires. Additionally, energy and power densities reached during charge by the NN setup are higher than the bulk configurations. Discharge energy and power densities were lower for this single-step process, excepting BB in ionic liquid that reached higher values during discharge; this could be attributed to polymer film over-oxidation during the process. To check cyclability, multiple-step charge/discharge transients were applied to the BB and NN configurations and media (Figure 2); thus, the stability of the E/t profile, through cycles, is studied.

**Figure 3:**
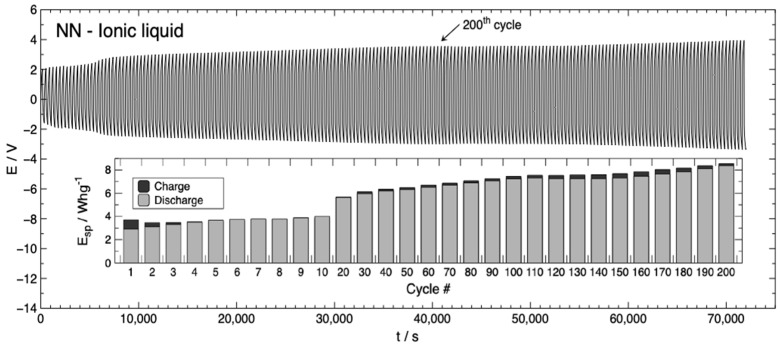
Multiple-step E/t charge/discharge transients of NN at ±2.3 μA, 400 cycles. Insert: Energy density vs. cycle number calculated for the first 200 cycles.

Multiple charge/discharge steps are applied to test device cyclability. The E/t charge and discharge profiles are more symmetrical in the NN configuration than those for BB in both media. Additionally, BB reaches higher and slightly increasing charge-potential values, possibly damaging the polymeric film after each step, and decreasing discharge/charge ratio. In the inserts, the energy density vs. cycle number is presented for both the charge and discharge steps. Energy density achieved for each step is higher (ca. 2 times) in ionic liquid compared with the aqueous medium. Additionally, energy density maximum values are somehow higher for NN than BB. However, the higher degree of reversibility accomplished by the NN setup compared with the BB is quite impressive. This reversibility is expressed as the energy density ratio between the discharge/charge steps for each cycle (see inserts Figure 2), and, thus, is a direct measure of system cyclability. Cyclability is not only better at NN configurations, but also increases after each cycle, producing a more stable device with longer lifespan. Finally, overall performance is not better for hybrid bulk–nano (BN) and nano–bulk (NB) electrode configurations (Figure S3, supplemental file) in both media, highlighting the importance of complete nanostructured device for optimum results. Most likely the higher energy density capacity reached by nano-structured electrodes would indirectly affect the behavior of the other bulk electrode. Since this NN configuration provided best performance, especially in ionic liquid, cyclability was further tested up to 400 cycles at ±2.3 μA charge (Figure 3).

At ±2.3 μA NN present great cyclability performance. Around cycle 200 the charge/discharge process generates a potential almost twice as great as that of the first cycles. This behavior may be attributable to the polarization of the electrodes when applying the electrochemical perturbation [30]. Meanwhile, the respective charge and discharge energy density vs. the number of cycles (Figure 3, insert) shows quite similar densities in both processes, proving good reversibility and longer device’s lifespan, maintaining of 98% discharge/charge performance. Nevertheless, a slight energy density increase vs. cycle is observed, attributable to the increase in charge-potential in each cycle. Table 2 present characteristics of batteries, designed with PEDOT or PPY layers, reviewed from the literature, compared to the outcomes obtained in the present report.

There are several reports in literature regarding the application of conducting polymers in battery design [5,6,14,15]. Nevertheless, one should take notice on different characterization methods in order make fair comparisons. For instance, studies considering battery characterizations made after solely cyclic voltammetry outcomes were discarded since, in this case, outcomes depend highly on voltammogram shape and symmetry. In comparison of the reviewed studies on Table 2, it should be noticed that the battery characteristics of NN configuration of this work far exceed the results from other authors in terms of specific capacitance, energy, power, and cyclability.

### 3.2. Solar Rechargeable Battery Characterization

**Figure 4:**
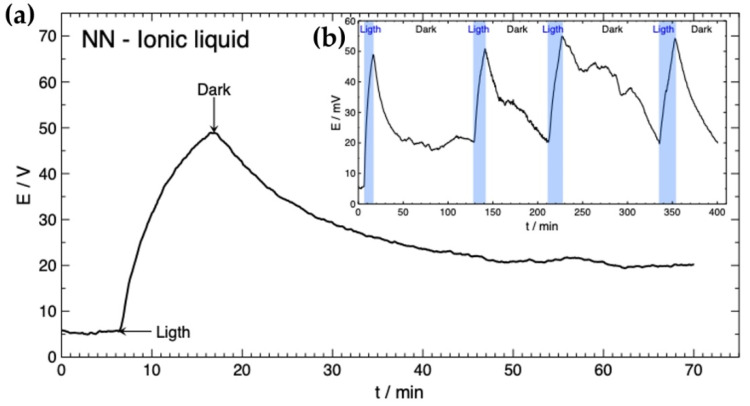
E/t transient of ITO|TiO_2_|N3|PEDOTnw|BMIMPF_6_|PPynw|ITO in light and dark conditions (**a**) Single-step and (**b**) Multiple-step.

The great performance of the NN device as a rechargeable energy device inspired the use of a nanostructured conducting polymer setup in a solar energy-based rechargeable battery. An ITO|PPynw cathode and an ITO|TiO_2_|PEDOTnw photo-anode [27] are assembled including a N3 dye layer under the described conditions, producing the solar energy rechargeable battery ITO|TiO_2_|N3|PEDOTnw|BMIMPF_6_|PPynw|ITO. The measurements of this device are carried out in two steps: (i) photo-recharge and (ii) discharge, independent of each other (Figure S4a, supplementary information). Single and multiple-step photo-recharge/discharge transients are presented in Figure 4.

In Figure 4a a single-step E/t transient of the first photo-recharge/discharge cycle can be seen and, during the first 10 min under the 100 mW cm^−2^ white light illumination, an increase of 45 mV in its potential is observed (Figure S4b, Supplementary Information). Subsequently, the photo-recharge system was disconnected (Figure S4c, Supplementary Information) and the discharge system connected, observing that the potential of the system decreases until reaching a stable potential around 20 mV for approximately one hour. This photo-recharge/discharge process was reproduced during four cycles (Figure 4b), similar to analogous systems found in the literature [26]. Each photo-charge step achieved a maximum 50–55 mV of charge potential, while, during discharge, potential decreases to stable value of 20 mV; this accounts ca. 35 mV photo-recharge potential difference or open circuit voltage. Accordingly, the charge reaction scheme is as follows [26]:

(4) $$N3+hv\to N3^{*}$$

(5) $$N3^{*}+TiO_{2}\;\to \;e+N3^{*+}$$

(6) $$N3^{*+}+PEDOTnw+xPF_{6}^{-}\;\to PEDOTnw^{x+}\cdot xPF_{6}^{-}+N3$$

(7) $$PPynw^{x+}\cdot xPF_{6}^{-}+xe\to PPy+xPF_{6}^{-}$$

With Equations (4)–(6) taking place at the anode and (7) correspond to electron-storage at the cathode. The overall charge reaction is as follows:

(8) $$PEDOTnw+xPF_{6}^{-}\;\to PEDOTnw^{x+}\cdot xPF_{6}^{-}+xe$$

During discharge, in the dark, the reaction scheme is as follows:

(9) $$PPy+xPF_{6}^{-}\;\to \;PPynw^{x+}\cdot xPF_{6}^{-}+xe$$

(10) $$PEDOTnw^{x+}\cdot xPF_{6}^{-}+xe\to \;PEDOTnw+xPF_{6}^{-}$$

With (9) and (10) occurring at the cathode and anode, respectively. The overall discharge reaction is (11), as follows:

(11) $$PEDOTnw^{x+}\cdot xPF_{6}^{-}+PPy\to \;PEDOTnw+PPynw^{x+}\cdot xPF_{6}^{-}$$

Thus, *PEDOTnw* reduction and *PPynw* oxidation processes are induced by light increasing OCV by ca. 35 mV.

Table 3 shows a comparison of different studies considering the use of some of the materials described in present report in the design of solar-energy rechargeable devices. It should be noted that characterization and manufacturing conditions from reviewed articles are quite different from those of the present study. When comparing the studies, it is possible to observe that the open circuit potential of present work is substantially lower than the studies described by Takshi et al. [32] and Lei et al. [33]. This can be attributed to the concentration present in the proposed photoanode, an important factor for generating the photo-recharge. On the other hand, electrode area (0.21 cm^2^) in the present study is quite low compared with those presented in the other works, where 0.5 cm^2^ stands out in the Takshi et al. report [32].

Nevertheless, the behavior observed in open circuit potential (OCV) measurement under dark and illumination conditions is a promising outcome to proceed with in electrode optimization experiments, as a function of coulombic efficiency in future studies. Finally, the nanostructured electrodes from present report have shown impressive performance after characterization as a reversible battery device. It has highly surpassed the performance in terms of specific charge, energy power, and cyclability compared with similar devices in the literature. In addition, due to the semiconducting properties of components, the device is also potentially useful as a solar-rechargeable battery. The device performance could be further enhanced if some experimental variables are optimized, such as the nanowire size and the use of illumination during electropolymerization step, as has been made by other authors [26]. Therefore, it can be assured that the described system possesses many potential and further optimization studies will be pursued.

## 4. Conclusions

The methodology is suitable for direct electro-deposition of PEDOT and PPy nanowires. It has been demonstrated that these electrodes can used in the manufacture of a battery with overall good performance if compared with reports from other authors. The NN configuration greatly enhances characteristics such as specific capacity, energy, and density. Device cyclability is also better compared with reports with similar materials found in the literature, especially when ionic liquid is employed, from the different combinations.

In short, it has also been demonstrated that it is possible to generate the photo-recharge of an energy storage device by modifying its anode through the incorporation of TiO_2_ and a dye, transforming it into a photo-anode, which it allows an effective generation of current. The nanostructured solar-rechargeable battery device performance could be further improved by controlling preparation conditions, such as the use of illumination during the electrochemical polymerization of conducting polymer nanowires. Nevertheless, the potentiality of the device, regarding capacity, repeatability, reversibility, and small size is very promising.

## Figures and Tables

**Table 1:** Specific total capacity, energy density, and power density for different battery configurations and media.

Battery	$Q_{tot}$	$E_{spec}$		$P_{spec}$	
	Ah g^−1^	Wh kg^−1^		W kg^−1^	
		Charge	Discharge	Charge	Discharge
BB-Aqueous	148	423	153	8469	3067
NN-Aqueous	2471	3794	860	75,884	17,196
BB-Ionic liquid	184	655	153,333	13,109	3,066,667
NN-Ionic liquid	2868	7901	1182	158,027	23,645

**Table 2:** Comparison of batteries characteristics for other reports with similar materials.

Battery Configuration	I	$Q_{tot}$	$E_{spec}{\;}^{a}$	$P_{spec}{\;}^{a}$	Cycle n°/Stability	Ref
	A g^−1^	Ah g^−1^	Wh kg^−1^	W kg^−1^		
Al|EMImCl-AlCl_3_|PEDOT	0.03	179	233	146	100/94%	[14]
SS|E-AVNF|Zn	0.50	326	276	16,000	1000/94%	[5]
NiO/PEDOT|EC/DEC + LiPF_6_|Li	0.72	650	n/a	n/a	50/80%	[6]
Sn|PPy|Cu	n/a	942	n/a	n/a	50/47%	[15]
ITO|TiO_2_|PEDOTnw|BMIMPF_6_|PPymw|ITO	57.4	2868	1075	21,495	200/98%	This work

Key: I (applied current per mass of active material); ^a^ (calculated after discharge process); EMImCl-AlCl_3_ (1-ethyl-3-methylimidazolium chloride-aluminum chloride); SS (stainless steel foil); E-AVNF (PEDOT intercalated ammonium vanadate nanofiber); NiO/PEDOT (nickel oxide film with PEDOT deposit); EC/DEC (ethylene carbonate and diethyl carbonate mix); BMIMPF_6_ (1-butyl-3-methylimidazolium hexafluorophosphate).

**Table 3:** Comparison of solar rechargeable batteries for various reports with similar materials.

Active Layer Material		Medium	OCV/mV	Ref
Anode	Cathode			
TiO_2_|PEDOT ^a,b^	PPy ^c^	LiClO_4_ + TBP in PC	510	[26]
ZnTpp|PEDOT:PSS	Carbon paper	MV in TB	430	[32]
N749|PEDOT:PSS	Carbon paper	MV in TB	198	
TiO_2_|N719 ^a^	Hydrogen storage alloy ^d^	LiClO_4_ + PEDOT-modified Nafion^®^	700	[33]
TiO_2_|N3|PEDOTnw	PPynw	Ionic liquid	35	This work

Key: TBP (4-tertiary-butylpyridine); PC (propylene carbonate); ZnTpp (5,10,15,20-tetraphenyl-21 h,23 h-porphine-Zn); PSS (Polystyrene sulfonate); N749 (triisothiocyanato-(2,2′:6′,6′′-terpyridyl-4,4′,4′′-tricarboxylato) ruthenium(II) tris(tetra-butylammonium)); MV (Methylviologen); TB (Tris buffer); N719 ammonium salt of N3 dye; ^a^, TiO_2_ coating using titanium oxide paste sintered at 500 °C; ^b^, PEDOT layer made through photoelectrochemical polymerization method; ^c^, PPy layer made through electrochemical polimerization method; ^d^, MmNi_3.6_Co_0.75_Mn_0.35_Al_0.3_.

## Data Availability

Not applicable.

## Supplementary Materials

The following are available online at https://www.mdpi.com/article/10.3390/polym13244375/s1, Figure S1: SEM micrograph of (A) ITO|TiO_2_|PEDOT-nw and (B) ITO|PPy-nw, Figure S2: Single-step E/t charge/discharge transients registered at different charge currents in aqueous and ionic liquid media, Figure S3: Multiple-steps E/t charge/discharge transients registered at ±5 μA for nano–bulk (NB) and bulk–nano (BN) electrode configurations in different media. Insert: Energy density calculated for each charge/discharge cycle, Figure S4: Configuration (a) and operating mechanisms of ITO|TiO_2_|N3|PEDOTnw|Ppynw|ITO solar-energy-rechargeable battery during photo-charge (b) and discharge (c) processes.
