# Supplementary material for: Charge Storage and Solar Rechargeable Battery Devices Based on Electrodes Electrochemically Modified with Conducting Polymer Nanowires
Source: Polymers (Basel). 2021 Dec 14;13(24):4375. doi: 10.3390/polym13244375 (PMC8709196; doi:10.3390/polym13244375)
Supplement: Supplementary file 1 [file polymers-13-04375-s001.zip › polymers-1488549-supplementary.pdf]

SUPPLEMENTARY INFORMATION

Charge Storage and Solar Rechargeable Battery Devices Based on Electrodes Electrochemically Modified with Conducting Polymer Nanowires

Andrés Mauricio Ramírez [1], Manuel Alejandro Gacitúa [2], Fernando Raúl Díaz [3] and María Angélica del Valle [3,*]

[1] Laboratorio de Electroquímica y Materiales Aplicados, Centro de Nanotecnología Aplicada, Facultad de Ciencias, Universidad Mayor, Camino La Pirámide 5750, Huechuraba-Santiago 8580745, Chile; andres.ramirez@umayor.cl
[2] Facultad de Química y Biología, Universidad de Santiago de Chile, Av. L.B. O'Higgins 3363, Santiago 7254758, Chile; manuel.gacitua@usach.cl
[3] Laboratorio de Electroquímica de Polímeros, Pontificia Universidad Católica de Chile, Av. V. Mackenna 4860, Macul, Santiago 7820436, Chile; fdiaz@uc.cl
* Correspondence: mdvalle@uc.cl

Figure S1

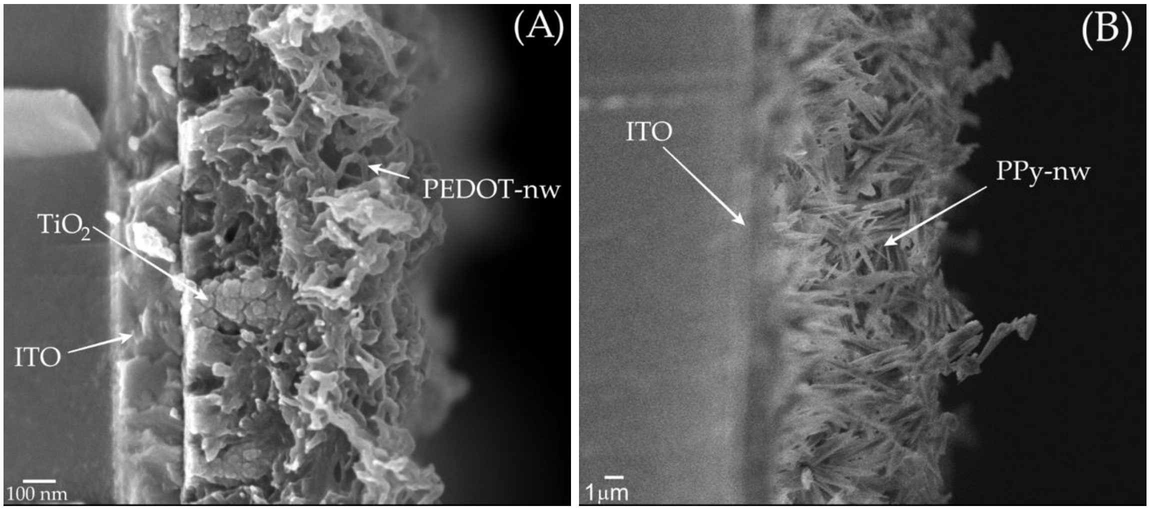

Figure S1 SEM micrograph of (A) ITO|TiO_2|PEDOT-nw and (B) ITO|PPy-nw.

Figure S2

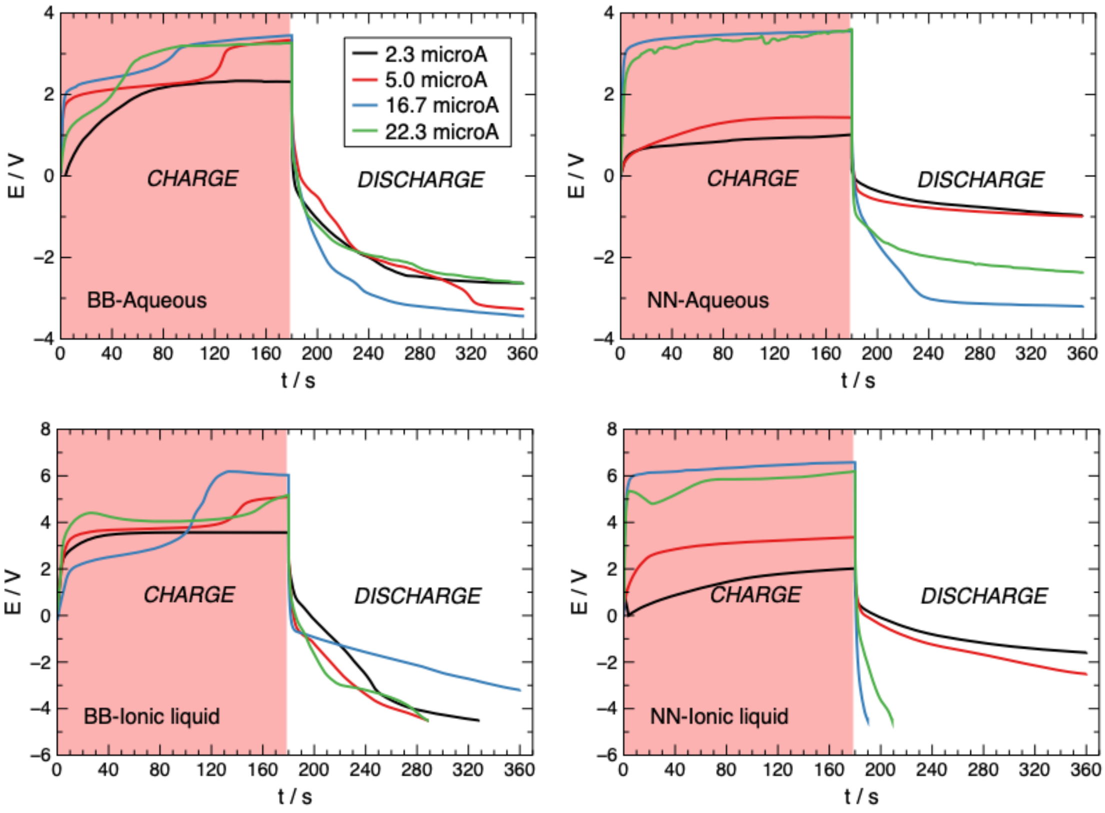

Figure S2. Single-step E/t charge/discharge transients registered at different charge currents in aqueous and ionic liquid media.

Figure S3

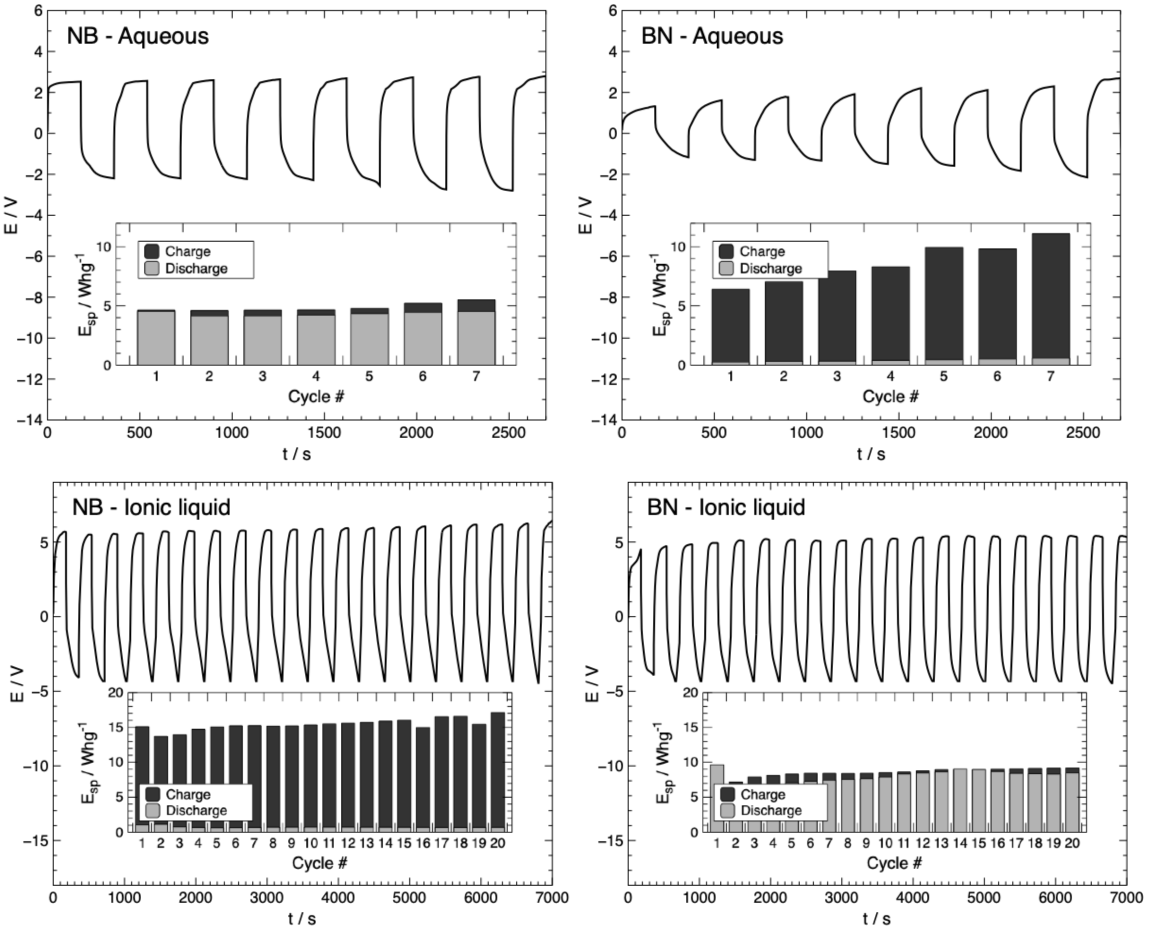

Figure S3. Multiple-steps E/t charge/discharge transients registered at ±5μA for nano-nulk (NB) and bulk-nano (BN) electrode configuration in different media. Insert: Energy density calculated for each charge/discharge cycle.

Figure S4

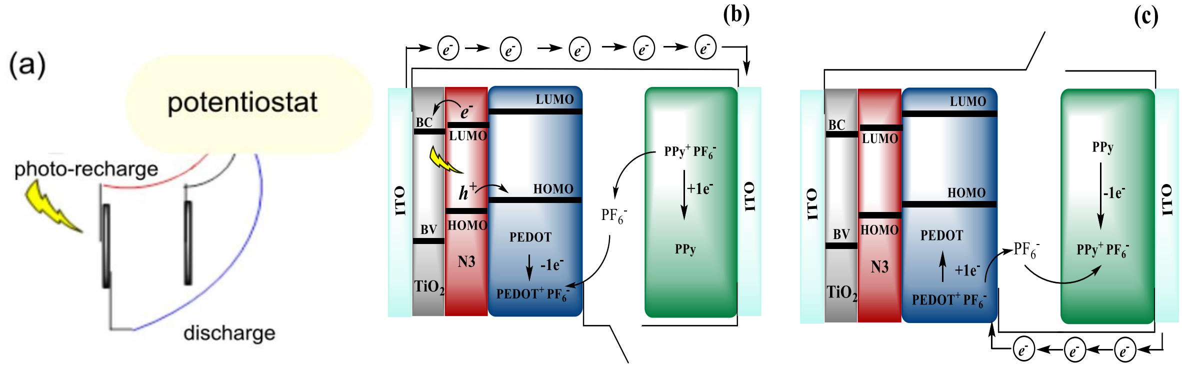

Figure S4. Configuration (a) and operating mechanisms of ITO|TiO_2|N3|PEDOTnw|Ppynw|ITO solar-energy-rechargeable battery during photocharge (b) and discharge (c) processes.
